# What do we talk about when we talk about rhythm?

**DOI:** 10.1371/journal.pbio.2002794

**Published:** 2017-09-19

**Authors:** Jonas Obleser, Molly J. Henry, Peter Lakatos

**Affiliations:** 1 Department of Psychology, University of Lübeck, Lübeck, Germany; 2 Brain and Mind Institute, Department of Psychology, The University of Western Ontario London, Ontario, Canada; 3 Cognitive Neuroscience and Schizophrenia Program, Nathan Kline Institute, Orangeburg, New York, United States of America; 4 Department of Psychiatry, New York University School of Medicine, New York, New York, United States of America; New York University, United States of America

Neural oscillations align to external stimulus rhythms, such as the recurring onsets in rhythmic sequences, via neural entrainment—that is, adjustment to the oscillation’s phase and period. As such, measures of neural phase coherence or “phase concentration” can inform us about the precision of entrainment. Entrainment of ongoing neural oscillations is considered a potent mechanism that the brain utilizes to generate temporal predictions and to aid active perception, at least in sensory cortices. However, as attempts to falsify or question this hypothesis are scarce, the idea that temporal predictions are instantiated in neural oscillatory entrainment should not be set in stone just yet.

For this reason, it is very important that a recent study by Breska and Deouell, published in *PLOS Biology* [1], set out to determine whether phase concentration at the time of a critical target stimulus would also be observed in instances when stimulus sequences are non-rhythmic, but targets are nonetheless temporally predictable [2]. Indeed, temporal predictions can certainly be formed on the basis of non-rhythmic information, such as the passage of time itself [3]. Breska and Deouell propose a “memory-based” prediction mechanism that would not be instrumented by entrainment but instead might utilize alternate, interval-based timing mechanisms [4,5]. While the study raises very important questions, logical and methodological considerations have motivated us to lay out a short guide for future experiments that aim to demonstrate oscillatory entrainment or the lack thereof. Throughout, we support our arguments with simulations and supplemental audio, which can be found at https://osf.io/phs6e/.

Breska and Deouell aimed to assess behavioral and electrophysiological measures in contexts that vary in their rhythmicity, with the goal of exploring the bounding conditions of neural entrainment as well as the neural mechanisms that might support temporal predictions when entrainment is not possible. The trouble with examining neural mechanisms in contexts with varying rhythmicity is that we are still lacking a good working definition of rhythm. What does “rhythm” mean to a human or non-human brain, and to a perceiver more generally, and how variable does a sequence of events need to be so that our brains will cease to register it as rhythmic? Many researchers seem to use “rhythm” to refer to isochrony, that is, strict regularity, and contrast this with situations in which sequences are composed using variable stimulus onset asynchronies (SOAs; e.g., “repeated-interval” and “random” conditions in Breska and Deouell’s study).

However, there are many types of sequences that might be perceived as rhythmic despite being decidedly aperiodic and having relatively high variance in terms of the intervals making up the rhythm. For example, metrical musical rhythms comprising intervals of different sizes (e.g., quarter notes, half notes, and dotted half notes) can give rise to a sense of regularity at a rate that is not actually represented by any of the intervals in the sequence (that is, at the whole note level). In fact, many previous studies on neural entrainment did not even utilize the strictly isochronous sequences presented here as a hallmark of rhythm but instead allowed for considerable jitter between single stimuli (e.g., [6–9]).

Empirically, rhythmicity can be assessed best by asking individuals whether they perceive a sequence as rhythmic. To this end, we transferred Breska and Deouell’s sequences to the auditory domain (arguably the more natural domain for assessing rhythm and entrainment) in an exercise that was quite informative: it turns out that their “repeated-interval” condition, conceived to reduce rhythmicity, feels very rhythmic indeed (see supplemental audio at https://osf.io/phs6e/). Theoretically, rhythmicity can be assessed by attempting to align (that is, to entrain) a sinusoidal oscillation to a stimulus sequence (Fig 1), as Breska and Deouell have also done: if the oscillation aligns to the sequence such that its relative phase is sufficiently non-random relative to stimulus onsets, the sequence should be considered rhythmic from the perspective of the underlying oscillator. Our attempts to do so suggest that the degree of jitter employed by Breska and Deouell, even in their “random” condition, would still allow for successful neural entrainment (Fig 1C). We note that Breska and Deouell also report results of such a simulation and also show that, in particular for the long SOA condition, their “random” stimulation would have led to successful oscillatory entrainment.

**Fig 1:**
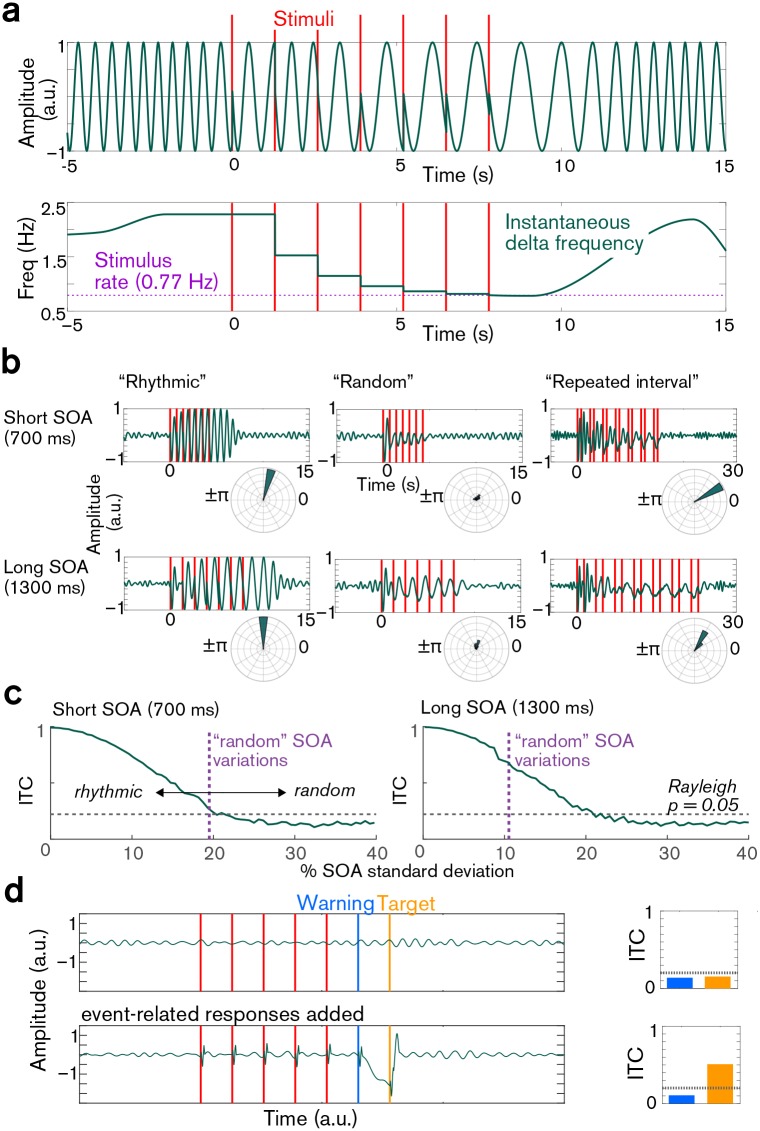
Pitfalls of “oscillatory investigations” into rhythm processing. **(a)** A 20-second-long snippet of simulated delta oscillation illustrating the properties of the model used to investigate the effects of task structure on the delta inter-trial coherence measure. The top trace shows a simulated ongoing delta oscillation that becomes entrained by the stimulus onsets, indicated by vertical red lines. The bottom trace is the time course of the instantaneous frequency of the delta oscillation; the horizontal line marks the stimulus rate. The ongoing oscillation had a default mean frequency of 1.7 Hz (with a 20% standard deviation of 0.34 Hz; Gaussian frequency distribution). Upon stimulus presentation, the oscillation was reset, and the period was sequentially adjusted to the frequency halfway between the current ongoing frequency and the SOA of the preceding 2 stimuli. **(b)** Simulated oscillatory entrainment by the stimulus structures employed by Breska and Deouell [1]. As the delta waveforms averaged across 100 instances of stimulus sequences consisting of 7 stimuli illustrate, delta phase across trials is not random, but rather delta oscillations are aligned to the stimuli, despite some randomization (for paradigm details see Breska and Deouell [1]). Indeed, ITC values measured at the last stimulus of each train (target in the Breska and Deouell study) were all significant (all *P* < 10^−5^), as the respective polar histograms next to each panel illustrate (covering a range of 0%–100% of all delta phases). **(c)** The effect of SOA randomization on phase concentration. Simulated ITC values at target onset for differing degrees of uniform variation in SOA. The x-axis displays percentage SOA standard deviation; the vertical dotted lines represent the actual SOA variation used by [1]. These both fall into the rhythmic category indicated by significant ITC values (horizontal dotted line, Rayleigh *P* = 0.05 for 100 trials, corrected for multiple comparisons). **(d)** A theoretic scenario in which ongoing oscillations are not reset or entrained by stimuli, e.g., when presented stimuli are being ignored [10]. On top, no event-related potentials are present, and delta phases measured with the phase estimation method used by Breska and Deouell are random across the 100 stimulus train presentations as the non-significant ITC values measured at the time of the second to last (Warning) and last (Target) stimuli show (bar plot on the right). On the bottom, we added event-related responses including a CNV component following the Warning stimulus and a P3 component following the Target (like responses in the Breska and Deouell study). In this case, even when phases of underlying delta oscillations are completely random, significant ITC is detected due to the large amplitude ERP components flanking the target. ITC related to the warning stimulus is still non-significant, similar to Breska and Deouell’s results (Fig 5B in [1]). Note that the amplitude of the CNV component was twice the amplitude of ongoing delta in our simulations, and that evoked response-related phase bias depends on the ratio of amplitudes as well as on the shape of the response [11,12]. **Abbreviations:** CNV, contingent negative variation; ERP, event-related potential; ITC, Inter-trial [phase] coherence; SOA, stimulus onset asynchrony.

An orthogonal issue contributing to Breska and Deouell’s largely similar phase-concentration results across “rhythmic” and “repeated-interval” conditions is the presence of large-amplitude contingent negative variation (CNV) and P3 components flanking the targets, which likely distorted the phase measures and resulted in artificially high phase concentrations (Fig 1D). This suggestion (also put forward by Breska and Deouell) was confirmed by our simulations and accurately predicted lower phase concentration at the time of the warning signal compared to the time of the target, which would not be predicted by neural entrainment alone (Fig 5B of Breska and Deouell [1]). The bottom line here has already been discussed at length in the field: phase concentration measures do not just index oscillations—they index the entire underlying signal—and therefore should be interpreted with utmost caution.

Nonetheless, to conclude that “phase concentration and alignment of slow EEG [electroencephalogram] oscillations are not a signature of rhythmic entrainment” ([1]; as in the title of their paper) is worrisome: Breska and Deouell seem only to consider the possibility that entrainment measures might not be good indicators of entrainment. Potentially, however, their manipulation simply did not vary the intended independent measure (that is, rhythmicity) to a conclusive degree.

Our reservations notwithstanding, Breska and Deouell’s paper is an important reminder of how little we actually know about the way(s) in which rhythmic—or otherwise predictable—patterns in our environment are utilized by behaving organisms. Accordingly, a hallmark contribution of their paper is to demonstrate the behavioral costs (in terms of response times) that can arise for a behaving organism from entrainment to a strictly periodic stimulus.

Let us conclude by briefly outlining requirements of an experiment that we think would settle the important questions raised by Breska and Deouell more conclusively. First, the experiment would need to manipulate a perceiver’s ability to predict the precise timing of upcoming, behaviorally relevant events by truly varying rhythmicity. That is, the presence of rhythmicity should be empirically verified by asking for information about the (non-)rhythmic percept. One particularly promising approach is to manipulate meter: rhythms with different degrees of perceived rhythmicity can be constructed by reordering the exact same interval set, creating stimuli with identical statistical properties but different degrees of perceived regularity [13]. Such stimuli allow for a rigorous test of the effects of rhythmicity on neural entrainment.

Second, we suggest that unpredictable, near-threshold targets avoid as much as possible the contamination by large CNV and P3 components that can distort phase-concentration measures. Inevitably, however, testing a memory-based prediction mechanism is contingent on a subject’s ability to predict when the target will occur—a process that can hardly escape large transient evoked responses like the CNV and so may be better studied using a psychophysically sound behavioral paradigm. It remains to be conceded, however, that it is far from trivial to arbitrate between a (theoretically and empirically thus far substantiated) entrainment account on the one hand, and a potentially more parsimonious view that would subsume both periodic and aperiodic predictions but might challenge entrainment on the other.

So, what do we talk about when we talk about rhythm? As we have laid out here, regularity manipulations themselves need to be of sufficient magnitude and quality to modulate both percepts of rhythmicity and entrainment of a (neural or other) oscillator. Even then, however, our beloved tools for dissecting rhythmic and non-rhythmic processes in the neural domain can be turned into rusty blades by notorious interpretational problems. This should encourage us to humbly spell out our predictions in those domains where rhythm truly resides: in perception and behavior [14].

## Abbreviations

CNV: contingent negative variation
ERP: event-related potential
ITC: inter-trial [phase] coherence
SOA: stimulus onset asynchrony
