# Correction: What do we talk about when we talk about rhythm?

**DOI:** 10.1371/journal.pbio.1002615

**Published:** 2017-11-01

**Authors:** Jonas Obleser, Molly J. Henry, Peter Lakatos

The reference list is incorrectly numbered. References 12, 13 and 14 are incorrectly cited as references 10, 11 and 12 in the main manuscript. References 10 and 11 are incorrectly cited as references 13 and 14 in the main manuscript. The correctly numbered references are listed below and correspond to the correct citations.

## References

**1.** Breska A, Deouell LY. Neural mechanisms of rhythm-based temporal prediction: Delta phase-locking reflects temporal predictability but not rhythmic entrainment. PLoS Biol. 2017; 15(2):e2001665. https://doi.org/10.1371/journal.pbio.2001665 PMID: 28187128

**2.** Morillon B, Schroeder CE, Wyart V, Arnal LH. Temporal prediction in lieu of periodic stimulation. J Neurosci. 2016; 36(8):2342–7. https://doi.org/10.1523/JNEUROSCI.0836-15.2016 PMID: 26911682

**3.** Nobre A, Correa A, Coull J. The hazards of time. Curr Opin Neurobiol. 2007; 17(4):465–70. https://doi.org/10.1016/j.conb.2007.07.006 PMID: 17709239

**4.** Teki S, Grube M, Griffiths TD. A unified model of time perception accounts for duration-based and beatbased timing mechanisms. Front Integr Neurosci. 2011; 5:90. https://doi.org/10.3389/fnint.2011.00090 PMID: 22319477

**5.** Kotz SA, Schwartze M. Cortical speech processing unplugged: a timely subcortico-cortical framework. Trends Cogn Sci. 2010; 14(9):392–9. https://doi.org/10.1016/j.tics.2010.06.005 PMID: 20655802

**6.** Besle J, Schevon CA, Mehta AD, Lakatos P, Goodman RR, McKhann GM, et al. Tuning of the human neocortex to the temporal dynamics of attended events. J Neurosci. 2011; 31:3176–85. https://doi.org/10.1523/JNEUROSCI.4518-10.2011 PMID: 21368029

**7.** Lakatos P, Karmos G, Mehta AD, Ulbert I, Schroeder CE. Entrainment of neuronal oscillations as a mechanism of attentional selection. Science. 2008; 320(5872):110–3. https://doi.org/10.1126/science.1154735 PMID: 18388295

**8.** Stefanics G, Hangya B, Hernadi I, Winkler I, Lakatos P, Ulbert I. Phase entrainment of human delta oscillations can mediate the effects of expectation on reaction speed. J Neurosci. 2010; 30(41):13578–85. https://doi.org/10.1523/JNEUROSCI.0703-10.2010 PMID: 20943899

**9.** Keitel C, Thut G, Gross J. Visual cortex responses reflect temporal structure of continuous quasi-rhythmic sensory stimulation. NeuroImage. 2017; 146:58–70. https://doi.org/10.1016/j.neuroimage.2016.11.043 PMID: 27867090

**10.** O’Connell MN, Barczak A, Schroeder CE, Lakatos P. Layer specific sharpening of frequency tuning by selective attention in primary auditory cortex. J Neurosci. 2014; 34(49):16496–508. https://doi.org/10.1523/JNEUROSCI.2055-14.2014 PMID: 25471586

**11.** Lakatos P, Musacchia G, O’Connel MN, Falchier AY, Javitt DC, Schroeder CE. The spectrotemporal filter mechanism of auditory selective attention. Neuron. 2013; 77(4):750–61. https://doi.org/10.1016/j.neuron.2012.11.034 PMID: 23439126

**12.** Zhou H, Melloni L, Poeppel D, Ding N. Interpretations of frequency domain analyses of neural entrainment: periodicity, fundamental frequency, and harmonics. Front Hum Neurosci. 2016; 10:274. https://doi.org/10.3389/fnhum.2016.00274 PMID: 27375465

**13.** Grahn JA, Brett M. Rhythm and beat perception in motor areas of the brain. Journal of cognitive neuroscience. 2007; 19(5):893–906. https://doi.org/10.1162/jocn.2007.19.5.893 PMID: 17488212

**14.** Krakauer JW, Ghazanfar AA, Gomez-Marin A, MacIver MA, Poeppel D. Neuroscience needs behavior: Correcting a reductionist bias. Neuron. 2017; 93(3):480–90. https://doi.org/10.1016/j.neuron.2016.12.041 PMID: 28182904
